# Utrophin Up-Regulation by an Artificial Transcription Factor in Transgenic Mice

**DOI:** 10.1371/journal.pone.0000774

**Published:** 2007-08-22

**Authors:** Elisabetta Mattei, Nicoletta Corbi, Maria Grazia Di Certo, Georgios Strimpakos, Cinzia Severini, Annalisa Onori, Agata Desantis, Valentina Libri, Serena Buontempo, Aristide Floridi, Maurizio Fanciulli, Dilair Baban, Kay E. Davies, Claudio Passananti

**Affiliations:** 1 Istituto di Neurobiologia e Medicina Molecolare, Consiglio Nazionale delle Ricerche, European Brain Research Institute, Rome, Italy; 2 Italian Association for Cancer Research, Roman Oncogenomic Center, Rome, Italy; 3 Istituto di Biologia e Patologia Molecolari, Consiglio Nazionale delle Ricerche, Regina Elena Cancer Institute, Rome, Italy; 4 Department of Experimental Medicine, University of L'Aquila, L'Aquila, Italy; 5 Laboratory B, Regina Elena Cancer Institute, Rome, Italy; 6 Department of Physiology, Anatomy and Genetics, Medical Research Council Functional Genetics Unit, University of Oxford, Oxford, United Kingdom; Medical College of Georgia, United States of America

## Abstract

Duchenne Muscular Dystrophy (DMD) is a severe muscle degenerative disease, due to absence of dystrophin. There is currently no effective treatment for DMD. Our aim is to up-regulate the expression level of the dystrophin related gene utrophin in DMD, complementing in this way the lack of dystrophin functions. To this end we designed and engineered several synthetic zinc finger based transcription factors. In particular, we have previously shown that the artificial three zinc finger protein named Jazz, fused with the appropriate effector domain, is able to drive the transcription of a test gene from the utrophin promoter “A”. Here we report on the characterization of Vp16-Jazz-transgenic mice that specifically over-express the utrophin gene at the muscular level. A Chromatin Immunoprecipitation assay (ChIP) demonstrated the effective access/binding of the Jazz protein to active chromatin in mouse muscle and Vp16-Jazz was shown to be able to up-regulate endogenous utrophin gene expression by immunohistochemistry, western blot analyses and real-time PCR. To our knowledge, this is the first example of a transgenic mouse expressing an artificial gene coding for a zinc finger based transcription factor. The achievement of Vp16-Jazz transgenic mice validates the strategy of transcriptional targeting of endogenous genes and could represent an exclusive animal model for use in drug discovery and therapeutics.

## Introduction

Duchenne muscular dystrophy (DMD) is a severe muscle degenerative disease, which results from the absence of the cytoskeletal protein dystrophin. Becker muscular dystrophy (BMD) is a milder form of the disease, characterized by the presence of partially functional truncated forms of the dystrophin protein. No effective treatment for DMD/BMD is yet available [1]–[2]. Potential therapeutic approaches to DMD include the up-regulation of the utrophin gene, an ubiquitously expressed autosomal paralogue of dystrophin [3]. Utrophin displays about 80% homology with dystrophin and is able to perform similar functions. In particular, both the dystrophin and utrophin proteins bind members of the dystrophin associated protein complex (DAPC), forming a link between the extracellular-matrix and the intracellular actin cytoskeleton, that provides structural integrity to muscle fibers [4]–[5]. In adult muscle, utrophin is localized preferentially at the neuromuscular junction (NMJ) and at myotendinous junctions, while dystrophin is localized along the entire length of the sarcolemma [6]. However, in developing muscle, in regenerating muscle after injury and in mdx (mice dystrophin-deficient) skeletal muscle, utrophin is also found along the sarcolemma [7]. It is now well established that ectopic expression of utrophin prevents muscular dystrophy both in mdx mice and in utrophin-dystrophin deficient mice [8]–[13]. Therefore, many research groups are studying the possibility of up-regulating utrophin gene expression, either using pharmacological approaches or by trying to understand the “natural” mechanisms of increasing utrophin expression in developing, regenerating or pathological muscle [14]–[16]. To achieve utrophin up-regulation, our research group designed and engineered several zinc finger based synthetic transcription factors, capable of binding and activating transcription from the utrophin promoter “A” [17]–[18]. In the large class of proteins capable of binding DNA, zinc finger proteins have been preferred for the design of artificial transcription factors on the basis of their structural plasticity and modularity [19]–[21]. In particular, a “recognition code” that relates the amino acids of a single zinc finger to its associated subsite DNA triplet has been elaborated for several finger domains [22]–[30]. Multiple natural/synthetic zinc fingers assembled in tandem generate multifinger proteins that, when fused to the appropriate effector domain, can act as transcription factors and have a wide range of potential applications [31]. Significantly, zinc finger artificial transcription factors (ZF ATFs) can be used as therapeutics, for their ability to bind and re-program the expression of virtually any desired gene. Using the available “recognition code”, we previously engineered a gene named Jazz, encoding for a three-zinc finger peptide designed to specifically bind the 9 base pair DNA sequence 5′-GCT-GCT-GCG-3′ present in promoter “A” of both the human and mouse utrophin genes [32]. We have shown that the chimeric proteins, obtained by fusing Jazz with the appropriate effector domains, are able to drive the transcription of a test gene from both natural and synthetic utrophin promoters [17].

Here, we show that the ZF ATF “Vp16-Jazz” expressed in transgenic mice under the control of the myosin light chain (MLC1) promoter powerfully up-regulates its target gene “utrophin” at the chromosomal site. To our knowledge, this is the first example of a transgenic mouse expressing an artificial zinc finger transcription factor working on its endogenous target gene.

## Results

### Structure of the artificial Vp16-Jazz gene

**Figure 1:**
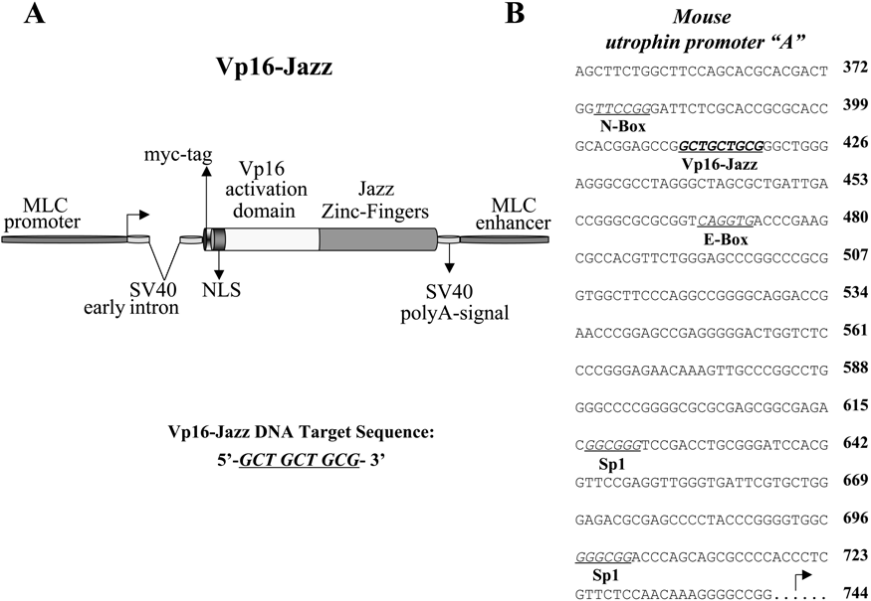
Vp16-Jazz and its DNA target sequence. A: Schematic representation of the Vp16-Jazz gene in the pMex-vector, used to generate transgenic mice. The 9 base pair long Vp16-Jazz DNA target sequence is indicated. B: The nucleotide sequence of the mouse utrophin promoter A. The Vp16-Jazz DNA target sequence is indicated in bold characters and underlined. The main transcription factor binding sites present in this promoter region are indicated.

We have previously shown that the chimeric proteins, obtained by fusing the Jazz zinc-finger gene with the appropriate transcriptional effector domain are able to drive the transcription of a test gene from both natural and synthetic utrophin promoters [17]. Now, we have engineered a novel Jazz-fusion protein named “Vp16-Jazz” in order to optimize its expression in transgenic mouse skeletal muscle. The Vp16-Jazz construct (Fig. 1A) contains the Myosin Light Chain (MLC) promoter/enhancer regions, an intron region derived from the Simian virus SV40, the strong transcriptional activation domain Vp16 from herpes simplex virus, the epitope tag myc and a nuclear localization signal (NLS) derived from SV40 early genes. The Vp16-Jazz protein specifically binds the 9 base pairs DNA sequence 5′-GCT-GCT-GCG-3′ present in the promoter region of both the human and mouse utrophin genes [32] (Fig. 1B).

### Vp16-Jazz expression in transgenic lines

**Figure 2:**
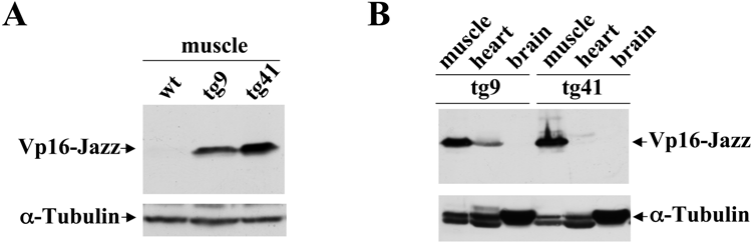
Muscle specific expression of Vp16-Jazz in transgenic mice. A: Western Blot analysis of total proteins extracted from the skeletal muscle of wild type (wt) and transgenic mice derived from two different founders (tg9 and tg41). The expression of Vp16-Jazz transgene was monitored by the anti-myc monoclonal 9E10 antibody. Detection of α−tubulin was used to normalize the amount of proteins. B: Western Blot analysis of total proteins extracted from the skeletal muscle, heart and brain of transgenic mice from families tg9 and tg41.

Using the above described Vp16-Jazz construct, several Fo transgenic mice founders were obtained. The expression of the transgene was analyzed by western blot on F1 positive mice using a monoclonal anti-myc antibody. Different levels of Vp16-Jazz expression were detected in skeletal muscle in several independent families. Figure 2A reports the level of Vp16-Jazz in transgenic families tg9 and tg41, that presented optimal transgene expression.

In order to verify the specificity of transgene expression in families tg9 and tg41, total protein extracts from muscle, heart and brain were analyzed in western blot. As shown in figure 2B the Vp16-Jazz is absent in brain, strongly present in skeletal muscle and slightly present in heart only in family tg9. Other tissues were also analysed, which confirmed muscle specific expression of Vp16-Jazz (data not shown).

### Vp16-Jazz and utrophin up-regulation

**Figure 3:**
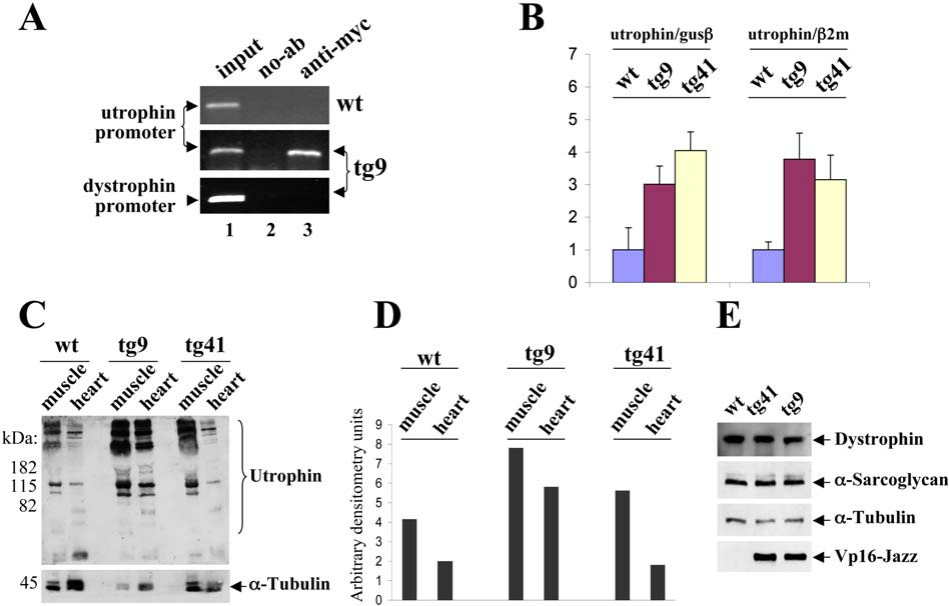
Vp16-Jazz and utrophin up-regulation. A: Vp16-Jazz chromatin immunoprecipitation, performed in skeletal muscle derived from wt mice and transgenic mice (family tg9) using myc monoclonal antibody/protein G-agarose beads or protein G-agarose beads as a control (no-Ab). Immunoprecipitates from each sample were analyzed for the presence of utrophin promoter by PCR. A sample representing linear amplification of the total input chromatin (input) was included (lane 1). As control, samples from transgenic mice were also tested for the presence of dystrophin promoter sequence. B: Real-time PCR analysis of the utrophin gene expression rate in Vp16-Jazz transgenic mice (tg9 and tg41) and control wt mice. The gene expression ratio between utrophin and β-glucoronidase (GUS) and β2-microglobulin (β2M) is shown as means±S.D. from three independent experiments performed in triplicate. C: Western blot of total protein extracts derived from skeletal muscle and heart from wt mice and Vp16-Jazz transgenic mice (tg9 and tg41) incubated with monoclonal antibody against utrophin. The same membrane was incubated with anti-α-tubulin monoclonal antibody for loading normalization. D: Relative utrophin expression of wt and transgenic mice (tg9 and tg41) was determined by densitometric analysis. E: Total protein extracts from skeletal muscle of wt and transgenic mice (tg9 and tg41) were subjected to immunoblotting to detect the expression levels of the dystrophin and α-sarcoglycan proteins. The anti-α-tubulin and anti-myc antibodies were used to normalize the protein content and to test the Vp16-Jazz transgene expression respectively.

In order to characterize the biological activity of the Vp16-Jazz protein in transgenic mice chromatin immunoprecipitation experiments (ChIP) were performed in muscle to verify whether the Vp16-Jazz transcription factor binds its DNA target *in vivo* in the context of the utrophin promoter chromatin infrastructure. In both tg9 (Fig. 3A) and tg41 (data not shown) Vp16-Jazz is able to bind efficiently and specifically to its DNA target sequence (5′-GCT-GCT-GCG-3′) at the endogenous utrophin promoter chromosomal site. In the ChIP experiment dystrophin promoter/enhancer region was used as further specificity control (Fig. 3A, bottom).

Next, we determined whether endogenous utrophin expression is effectively up-regulated by the artificial Vp16-Jazz protein. To this end, we performed a quantitative analysis of utrophin mRNA levels by real-time PCR. As shown in figure 3B, about 3 to 4 fold increase of utrophin expression was observed in transgenic mice compared to the control wild type (wt) mice. As shown, similar values were obtained using two different normalizing housekeeping-genes (β2-microglobulin and β-glucoronidase) .

To determine whether changes in the expression of utrophin mRNA were consistent with changes in the protein level a western blot analysis was performed using the anti-utrophin antibody. As shown in figures 3C and 3D, a consistent up-regulation of utrophin protein levels in skeletal muscle is observed in both transgenic families, tg9 and tg41, in comparison to wild type control animals. Notably, as expected, the up-regulation from the promoter region produces an increase of all the utrophin protein isoforms in the transgenic lines so far analyzed. Then we demonstrated that in both transgenic families, tg9 and tg41, and in wild type control mice dystrophin and alpha-sarcoglycan protein levels remain invariable in skeletal muscle (Fig. 3E).

### Skeletal muscle histological analysis

**Figure 4:**
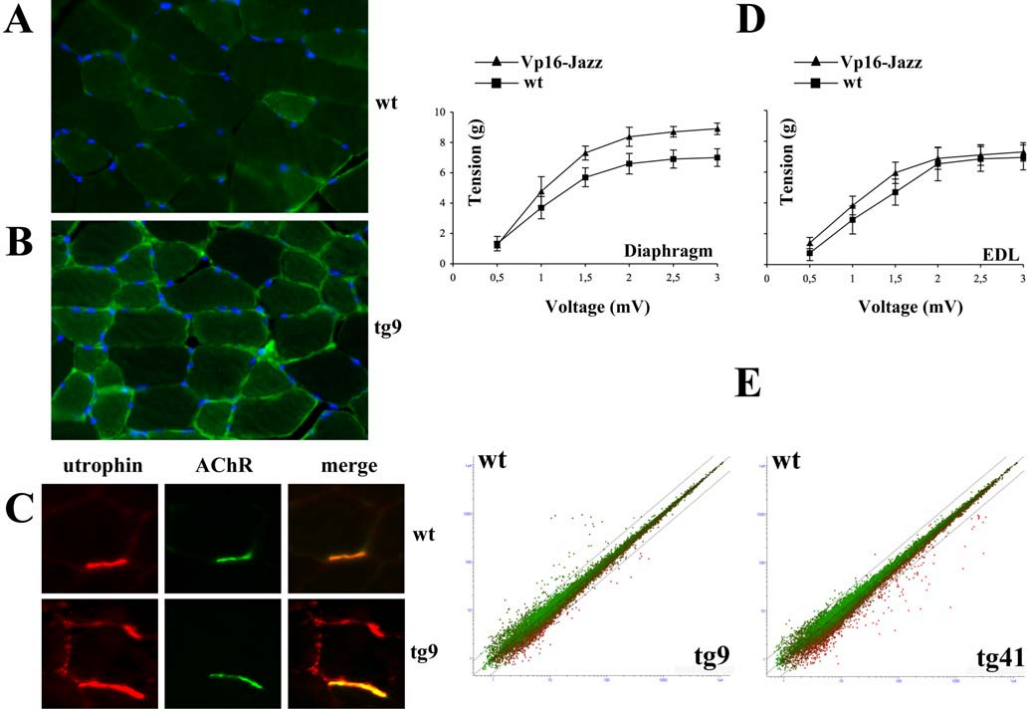
Effects of Vp16-Jazz in transgenic mice. Immunohistochemistry of Tibialis Anterior (TA) muscle derived from wt (panel A) and transgenic mice tg9 (panel B) stained with anti-utrophin antibody. Nuclei are counterstained with Hoechst 33258. C: TA from wt and transgenic mice tg9 were co-stained with anti-utrophin antibody and the α-bungarotoxin-Alexa Fluor to visualize the acetylcholine receptor (AChR) at the neuromuscular junctions. The anti-utrophin monoclonal antibody reveals an extra-synaptic distribution of utrophin only in transgenic mice. D: Relationship between contractile response (g) and stimulation frequency (Volts) in diaphragm and EDL muscle preparations obtained from wt, tg9 and tg41 mice. E: Scatter plots of the DEG with natural log transformed expression values averaged over the 4 replicates. The x–axis reports the mean of the replicates of transgenic mice, while the y-axis is the mean across the replicates of the control wt mice. Expression values are colour coded with red representing up-regulated genes in tg9/tg41 and green down-regulated genes compared to wild-type and lines on the graph set at 2-folds differential expression.

The histological analysis of the tibialis anterior (TA) stained with the utrophin antibody revealed a significant increase and consequent re-localization along the sarcolemma of utrophin protein in the transgenic mice tg9 (Fig. 4B) and tg41 (data not shown) compared to wt control animals (Fig. 4A). In order to further investigate utrophin re-localization, we stained TA with both anti-utrophin antibody and with Alexa Fluor 488 labeled α-bungarotoxin to visualize the acetylcholine receptors (AChR) at the neuromuscular junction. As shown in figure 4C the utrophin overexpression in transgenic mice tg9 causes a remarkable re-localization of utrophin protein along the sarcolemma in addition to its canonical localization at the synaptic regions.

### Mechanical response of isolated muscles

The contractile activity of muscles from two month old Vp16-Jazz transgenic mice, and wt control animals was measured. Isolated diaphragm and extensor digitorum longus (EDL) preparations of both hind limbs were examined *in vitro* by physiological assessment of muscle force. As shown in figure 4D, both diaphragm and EDL muscles from transgenic mice show a slight, but constant increase in muscle strength as compared to wild type littermates. The increase in muscle strength was similar between the tg9 and tg41 families.

### Microarray analysis

To test the wide range genome perturbation induced *in vivo* by the artificial zinc finger transcription factor Vp16-Jazz and consequent utrophin over-expression, microarray analysis was performed, comparing the gene expression profiles of the Vp16-Jazz transgenic tg9 and tg41 mice versus control wt mice. Figure 4E represents scatter plots of the natural log transformed expression values of all genes (22690) on the array. Differentially expressed genes (DEG) found in families 9 and 41 versus control wt mice are colour coded with lines on the graph set at 2-folds differential expression.

## Materials and Methods

### Constructs

The Jazz gene fused to a nuclear localization signal region and the Vp16 activation domain from the herpes simplex virus was sub-cloned into the pMEX mammalian vector containing the MLC1 promoter/enhancer [33].

### Generation of Jazz transgenic mice

The DNA fragment from pMEX-Jazz containing the MLC1 promoter/enhancer and the Jazz-TF as described above was purified using a Geneclean Kit (Q-BIOgene, MP Biomedicals Morgan Irvine, CA) and resuspended in injection buffer (10 mM Tris pH 7,5, 0.1 mM EDTA, 30 mM NaCl) at a concentration of 2 ng/ml.

BDF1 (C57Black6×DBA) mice were purchased from Charles River (Calco, Italy), housed under conventional, controlled standard conditions and sacrificed by carbon dioxide asphyxiation.

Transgenic mice were generated by microinjecting the transgenic DNA into the pronuclei of fertilized eggs derived from BDF1×BDF1 mating, following previously described standard procedures [34]. The transgenic lines were maintained by crossing founders (F0) and F1 homozygous mice with wild type BDF1 partners. F1 and F2 transgenic mice and their wild type littermates of age comprised between 2 and 3 months were used in all experiments.

### Mice Genotyping

Transgene integration in F0 (founders) and transmission in F1/F2 animals was checked by polymerase chain reaction (PCR) analysis. Genomic DNA was isolated from the tail following standard procedures. Briefly, tails were incubated overnight at 53°C in 0.5 ml of tail solution (50 mM Tris-HCl, pH 8, 100 mM EDTA, 1% SDS, 100 mM NaCl) supplemented with 500 µg of Proteinase K (PCR grade, Roche). DNA was extracted using phenol/chloroform, precipitated with isopropanol, and dissolved in TE buffer (10 mM Tris-HCl, pH 7.5, 1 mM EDTA). 1 µg of genomic DNA was amplified by PCR in 50 µl of reaction buffer containing 0.4 mM dNTPs, 2.5 units of *Taq* DNA polymerase (Invitrogen Corporation, Carlsbad, Ca) and 1 µM of the following primers: primer 1: 5′-GTC GCC CCC CCG ACC GAT GTC AGC-3′; primer 2: 5′-GGG CGA TCC AGG ATC CCC GGG AAT-3′. PCR reactions were carried out at 94°C for 1 minute, followed by 94°C for 1 minute, 62°C for 2 minutes, and 72°C for 2 minutes, for a total of 30 cycles.

### Immunoblotting

The following mouse monoclonal antibodies were used for biochemical analysis: anti-Myc (9E10 clone, hybridoma-conditioned medium), anti-utrophin and anti-dystrophin (NCL-DRP2 and NCL-DYS2 respectively, Novocastra, Newcastle, UK), anti-alpha-sarcoglycan (Ad1/20A6, Vector Laboratories, Burlingame, CA, USA) anti alpha-Tubulin (Sigma Corporation, St. Louis, Missouri, USA). For protein extraction, all frozen tissues were processed in lysis buffer (2% SDS, 5 mM EDTA) supplemented with 1 mM PMSF and a proteinase inhibitor cocktail (Complete™, Roche Diagnostics GmbH Mannheim, Germany), using a homogenizer (7 mm, OMNI International GLH). Homogenates were boiled for 10 minutes and clarified by centrifugation for 20 minutes at 12000×g. The Quant-iT Protein Assay Kit was used to measure protein concentration by a Qubit™ fluorometer (Invitrogen), according to the manufacturer's instructions. 25 µg of protein extract was electrophoresed through SDS-polyacrylamide gel (SDS-PAGE) under reducing conditions and transferred onto nitrocellulose membranes (Schleicher&Schuell). Blots were probed with a dual immuno-staining with appropriate primary antibody and horseradish peroxidase-conjugated secondary antibody (Cell Signaling Technology Inc. Danvers, MA USA). The immunoreactive bands were visualized by chemiluminescence (ECL plus; GE Healthcare Amersham Buckinghamshire, UK), according to the manufacturer's instructions.

### Immunohistochemistry

Tibialis anterior (TA) muscles from transgenic and littermate control mice were fixed for 2 hours in 4% formaldehyde and cryoprotected in phosphate buffer (PBS)-33% sucrose before storage at −20°C. Transversal sections (8 µm thick) were obtained by cryostate at −20°C (LEICA, CM1850UV) and placed onto polysine-coated microscope slides (Menzel Gmbh&Co). Sections were permeabilized in PBS-0.2% NP-40 for 15 minutes and blocked in 5% goat serum. Slides were incubated with anti-utrophin monoclonal antibody (Novocastra, NCL-DRP2; 1∶20 dilution) for 1 hour, washed thoroughly and incubated with Alexa Fluor 488 goat anti-mouse secondary antibody (Molecular Probes; 1∶500 dilution) for 40 minutes. Nuclei were detected by co-staining sections with Hoechst 33258 (1 µg/ml) (Sigma) for 1 minute. All incubation steps were carried out at room temperature. Stained specimens were examined by conventional epifluorescence microscopy (Olympus BX51; Tokyo, Japan). Images were captured using a digital camera at 40× magnification and merged using the IAS2000 software.

Unfixed transversal sections (8 µm thick) from TA of wt and transgenic mice were stained with the α-Bungarotoxin Alexa Fluor 488 conjugated (Molecular Probes), a specific ligand of Acetylcholine Receptor (AChR), for 40 minutes at 37°C. Stained specimens were first examined by epifluorescence microscopy and then fixed in 4% formaldehyde to perform the utrophin staining. The Zenon Mouse IgG Labelling Kit (Molecular Probes) was used to directly conjugate the anti-utrophin monoclonal antibody with the Alexa Fluor 594, according to the manufacturer's instructions.

### Preparation of Total RNA

Total RNA was isolated from skeletal muscle by homogenization of tissue in Trizol reagent (Invitrogen), according to the manufacturer's instructions. The RNA yield was quantified by a Qubit™ fluorometer using the Quant-iT assay kit (Invitrogen).

The quality of the RNA was assayed by the Agilent 2100 Bioanalyzer, using the RNA 6000 Nano Assay Kit (Agilent Technologies).

### Real-time PCR analysis

2 µg of total RNA was reverse transcribed using oligo (dT) _12–18_ primers and *SUPERSCRIPT II* (Invitrogen) in a final volume of 20 µl at 42°C for 50 minutes. Real-time PCR assays were performed in a 96-well format using the ABI Prism 7000 Sequence Detection System (Applied Biosystems, Foster City, CA). To obtain the utrophin gene expression rate the amount of target gene was normalized to that of the two housekeeping genes β2-microglobulin (β2M) and β-glucoronidase (GUS) to correct for the differences in the quantity of the cDNA present in the PCR reactions. Primers and probes for the target gene (UTRN) and for housekeeping genes were purchased as TaqMan Gene Expression Assays (AB). The PCR mixtures containing the cDNA template, the TaqMan Universal PCR master mix (AB), and the primers/probes in a final volume of 25 µl were analyzed in triplicate, using the following conditions: incubation at 50°C for 2 minutes, denaturation at 95°C for 10 minutes and then 40 cycles of amplification at 95°C for 15 seconds and 60°C for 1 minute. For each gene amplification, a standard curve was generated using serial dilutions (200, 40, 8, 1.6 and 0.32 ng) of cDNA from mouse wild type (negative control). The results were analyzed using Applied Biosystems analysis software. The data are expressed as the ratio between UTRN and β2M or GUS mRNA expression. A minimal number of 9 mice were analyzed for each category.

### Chromatin Immunoprecipitation (ChIP) Assay

For one ChIP assay reaction, 100 mg of skeletal muscle from transgenic and littermate control mice was chopped into small pieces and cross-linked in 1% formaldehyde at 37°C for 15 minutes. After wash with PBS, tissues were homogenized (7 mm, OMNI International GLH) in lysis buffer (1%SDS, 1%TritonX-100, 10 mM EDTA 50 mM Tris-Hcl pH8.1) supplemented with a proteinase inhibitor cocktail (Complete™, Roche), and incubated for 60 minutes at 8°C. Samples were sonicated to generate DNA fragments with an average length of about 600 bp, diluted 10 fold in lysis buffer and centrifuged for 10 minutes at 13,000 rpm at 8°C. Supernatants were pre-cleared with Protein G-Agarose (Invitrogen) absorbed with sheared salmon sperm DNA (sSS-DNA, Eppendorf AG Hamburg, Germany) and bovine serum albumin (BSA, Sigma). At this step, a portion of pre-cleared sample (∼5%) was stored and considered as “input” sample. Immunoprecipitation of Myc-tagged protein-DNA complexes was achieved by incubating the pre-cleared samples overnight at 4°C with the anti-Myc monoclonal antibody (9E10 clone) and with Protein G-Agarose/sSS-DNA for a further 20 minutes. A “no-antibody” immunoprecipitation was performed as a negative control. All samples were eluted with 1% SDS, 0.1 M NaHCO_3_ and the cross-links were reverted by overnight incubation with 0.2 M NaCl at 65°C. Proteins were digested with Proteinase K (Roche) and DNA was purified by phenol/chloroform extraction. The final DNA was subjected to PCR in 25 µl of reaction buffer containing 0.8 mM dNTPs, 2.5 units of *Taq* DNA polymerase (Invitrogen) and 1 µM of the primers specific for the mouse utrophin or dytrophin promoter respectively, designed as follows:

m-utro forward: 5′-GCACGCACGACTGGTTCCGGGATTC-3′, m-utro reverse: 5′-CTTTGTTCTCCCGGGGAGACCAGTC-3′. m-dystro forward: 5′-CTGTGGCAGCTTAAGGCTTGTTCCCA-3′, m-dystro reverse: 5′-CATGTCGCCCGAGTGTTATTCTGTGC-3′.

For utrophin promoter/enhancer amplification PCR reactions were carried out at 94°C for 45 seconds, 66°C for 30 seconds and 72°C for 30 seconds, for a total of 33 cycles. For dystrophin promoter/enhancer PCR reactions were carried out at 94°C for 45 seconds, 62°C for 30 seconds and 72°C for 30 seconds, for a total of 33 cycles.

### Mechanical response of isolated muscles

Contractile activity of muscles from two month old Vp16-Jazz male mice and non-transgenic male littermates, was examined *in vitro* by physiological assessment of the muscle force of isolated diaphragm and extensor digitorum longus (EDL) preparations of both hind limbs. The muscle preparations were suspended in a 20 ml bath of oxygenated Krebs solution, maintained at 37°C, stretched to a tension of 1.0 g and allowed to equilibrate for 30–60 minutes, changing the superfusion buffer every 15–20 min. The diaphragm and EDL muscles were directly stimulated using single stimulations (Electric Stimulatore Digit 3T, Lace Elettronica, Pisa, Italy), carried out with rectilinear pulses of 0.5 msec duration at 0.05–0.2 Hz, using variable voltage, until the supramaximal voltage was reached. The mechanical activity of the muscle was recorded isotonically (7006 isotonic transducer) by a strain-gauge transducer and displayed on a recording microdynamometer (Unirecord 7050, Basile, Milano, Italy).

### Microarray

Total RNA was extracted using Trizol (Invitrogen Corporation, Carlsbad, Ca) and further purified using the RNeasy mini kit (Qiagen GmbH, Germany) according to the manufacturer's instructions. RNA was quantified using NanoDrop ND-1000 and RNA quality was checked using the Agilent bioanalyzer 2100 (Agilent Technologies Santa Clara, CA). A total of 12 high density oligonucleotide mouse MOE430A arrays (Affymetrix Toronto, Canada) were used in this study. All the procedures and hybridization were performed according to the Genechip expression technical manual (Affymetrix) as previously reported [35]. Briefly, 5 µg of RNA was converted to double-stranded cDNA with Superscript II (Invitrogen) using a T7-(dT)_24_ primer (Affymetrix), followed by *in vitro* transcription to generate biotin-labelled cRNA probes. Fragmented cRNA (15 µg) was used in 300 µl hybridization cocktail containing spiked controls (Affymetrix), 0.1 mg/ml Herring Sperm DNA (Promega Madison, WI, USA), and 0.5 mg/ml acetylated BSA (Invitrogen). A 200 µl aliquot of this hybridization cocktail was used on each chip which was incubated at 45°C for 16 hours rotating at 60 rpm. Following hybridization, the arrays were processed using a Genechip Fluidics Station 400 according to the recommended protocols (EukGE-WS2v4, Affymetrix) of double-staining and post-hybridizations washes. Fluorescent images were captured using gene Array Scanner 2500 (Affymetrix). All the experiments were designed and the information was compiled in compliance with MIAME [36] guide lines to ensure that the microarray data can be correctly interpreted and independently verified.

### Microarray data analysis

Gene transcript levels were determined from data image files using algorithms in Gene Chip Operating Software (GCOS1.2, Affymetrix). Global scaling was performed to compare genes from chip to chip thus the average signal intensity of the arrays was set to the same target intensity (TGT = 100). The key assumption of the global scaling strategy is that there are few changes in gene expression among the arrays being analyzed. All the data quality controls were carried out and met the Affymetrix quality assessment guidelines. Data analysis was performed using Data Mining Tool (DMT 3.1, Affymetrix) and GeneSpring 7.2 (Silicon Genetics). Robust Multi-Array Average (RMA) expression measurement was also used with the help of probe sequence (GC content) information (GCRMA) [37] as implemented in BioConductor R statistics (www.bioconductor.org). Cell Intensity files were processed into expression values for all the 22,626 probe sets (transcripts) on each of these arrays following the normalization step. Differentially expressed genes (DEG) were selected if they passed the Welch t-test, parametric test, with variance not assumed to be equal, p<0.05 and at least 2-fold changes between any two of the age matched genotypes used in this study.

## Discussion

Over the past few years, novel designed zinc fingers with modified functions led to a variety of applications [29], [31]. ZF ATFs permit to alter expression profiles of genes related to disease, with the aim of treating pathologies like cancer and genetic diseases [38]–[40]. Our laboratory has been focused on creating several novel transcription factors based on modified Cys_2_-His_2_ zinc finger motifs fused to positive or negative regulatory effector domains [17]–[18], [41]–[42]. In particular, we engineered ZF ATFs with the aim of up-regulating the utrophin gene, a possible substitute for dystrophin in DMD patients. Many research groups are approaching functional recovery of dystrophic muscle with different strategies [43]–[46]. One of the main problem emerged to perform any dystrophin gene therapy consists in the extremely large size of its mRNAs, that exceeds the incapsidation limits of many viral vectors. Recently, the last generation of gutted adenovirus appears to be able to package very large cDNAs including dystrophin [47]. In addition dystrophin minigenes, isolated from patients affected by Becker Muscular Dystrophy can partially circumvent the problem of dystrophin mRNA large size. On the other hand, utrophin has the fundamental advantage of being a protein present in DMD patients and thus being well tolerated by the immune system of the host. Our strategy is to re-program the expression of endogenous utrophin by ZF ATFs. We previously demonstrated that the three zinc finger protein Jazz is capable of binding and activating transcription from the utrophin promoter “A” [17]. Then, in order to obtain utrophin up-regulation in an animal model system, we designed and engineered a transgenic construct, named Vp16-Jazz, containing the Jazz zinc finger sequence under the control of the muscle specific promoter of the myosin light chain. Here, we report that the transgenic mouse model expresses Vp16-Jazz exclusively in skeletal muscle, with the exception of a faint expression of the transgene in the cardiac muscle of family tg9.

Once the chromatin immunoprecipitation experiments demonstrated that Vp16-Jazz was able to bind efficiently and specifically *in vivo* the DNA target sequence at the endogenous utrophin promoter, Vp16-Jazz ability to re-program expression of utrophin was investigated. The increase of utrophin mRNA ranged from 3 to 4 fold in mice from different transgenic families as compared with their non-transgenic littermates. The changes in the expression of utrophin mRNA were consistent with the increase in the utrophin protein level demonstrated by western blot analysis and by immunohistochemistry in the skeletal muscle of transgenic lines. Moreover, western blot analysis revealed, as expected, that the up-regulation from the endogenous promoter region mimics the body's natural regulatory mechanism, resulting in the production of several different utrophin isoforms in the transgenic lines analyzed so far. Interestingly, the tg9 that showed leaky expression of the transgene in cardiac muscle also displays an enhanced level of utrophin in the heart. This finding, not only confirms the efficiency of the artificial Vp16-Jazz in up-regulating utrophin expression, but also could became useful in future studies considering that utrophin deficiency worsens cardiac contractile dysfunction present in dystrophin-deficient mdx mice [48].

Moreover Vp16-Jazz specific expression has a promising positive effect on muscular strength as demonstrated by *in vitro* analysis of the mechanical response of isolated muscles. The different contractile responses to electric stimulation between diaphragm and EDL can be easily ascribed to the different nature and fiber composition of these two muscle [49]–[50].

Next, the wide range genome perturbation induced *in vivo* by Vp16-Jazz and by the consequent utrophin over-expression was tested by microarray analysis. The analysis of the gene expression profiles of the transgenic tg9 and tg41 mice versus control wt mice revealed an irrelevant global transcriptional effect. On the other hand, microarray analysis doesn't allow to distinguish between the transcriptional effects due to Vp16-Jazz transgene and the effects on global transcription due to the overexpression of the target gene utrophin.

In conclusion, the main results of our work were: i) the demonstration that a synthetic transcription factor introduced *in vivo* is well tolerated and safe for the health of the transgenic animal both during development and in adult life; ii) Vp16-Jazz was able to recognize specifically its DNA target sequence on the utrophin promoter and to up-regulate utrophin in muscle fibers without significant non-specific global transcriptional effects; iii) Vp16-Jazz specific expression has a promising positive effect on muscular strength as demonstrated by *in vitro* analysis of the mechanical response of isolated muscles. Thus the transgenic mouse described here represents the first example of an animal that could be useful to validate the use of ZF ATF technology for human gene therapy.

In future work, these Vp16-Jazz transgenic mice will be cross-bred with mdx mice. The achievement of Vp16-Jazz and Vp16-Jazz/mdx mice represents an useful animal model for use in drug discovery and therapeutics.
